# The rumour spectrum

**DOI:** 10.1371/journal.pone.0189080

**Published:** 2018-01-19

**Authors:** Nicolas Turenne

**Affiliations:** Affiliation Université Paris-Est, LISIS, INRA, 77454 Marne-La-Vallée, France; Universite Toulouse 1 Capitole, FRANCE

## Abstract

Rumour is an old social phenomenon used in politics and other public spaces. It has been studied for only hundred years by sociologists and psychologists by qualitative means. Social media platforms open new opportunities to improve quantitative analyses. We scanned all scientific literature to find relevant features. We made a quantitative screening of some specific rumours (in French and in English). Firstly, we identified some sources of information to find them. Secondly, we compiled different reference, rumouring and event datasets. Thirdly, we considered two facets of a rumour: the way it can spread to other users, and the syntagmatic content that may or may not be specific for a rumour. We found 53 features, clustered into six categories, which are able to describe a rumour message. The spread of a rumour is multi-harmonic having different frequencies and spikes, and can survive several years. Combinations of words (n-grams and skip-grams) are not typical of expressivity between rumours and news but study of lexical transition from a time period to the next goes in the sense of transmission pattern as described by Allport theory of transmission. A rumour can be interpreted as a speech act but with transmission patterns.

## Introduction

Disinformation (or misinformation) is a human language phenomenon that has always existed based on a mechanism of spreading from mouth to ear [1, 2]. However, with regard to the Internet and recent quantitative methods, we can investigate it with an up-to-date analysis. In the past, the spread of rumours could only be by word of mouth. The rise of social media provides an even better platform for spreading rumours. As Metaxas [3] explains massive amounts of data are being created and circulated, and often there are individuals or bots trying to manipulate this data to promote their own agenda. But sharing information with others after an emotionally powerful event can be cathartic. Understanding various rumour discussions could help to design and develop technologies to identify and track rumours, or reduce their impact on society.

In psychology a rumour is a declaration that is generally plausible, associated with news, and is widespread without checking [2, 4]. Some famous rumours are the urban legend “rue des Marmousets” in Paris where a barber and a pastry chef made cake trade based on human flesh in XVth century, or the disappearance of young girls in fitting rooms inside Jewish shops in the town of Orleans (France) in 1969 [5]. According to Gaildraud [6], a rumour is an informal noise that exists, persists, becomes evanescent and disappears as fast as it appeared. The definition of rumour is vague, such as one or several pieces of information that move around by individuals and/or the Internet. In the social sciences, rumouring behaviour is analysed as a social process of collective sense-making through which individuals can understand situations characterised by high levels of uncertainty, anxiety and a lack of official news. Classical social science research proposed two important ways of understanding rumour prevalence: (1) in terms of the amount of rumour-related information present in the environment, and (2) in terms of the number of individuals who have encountered or heard a particular piece of information. However, much of this very early work suffers from a lack of empirical support.

Ongoing research on the spread of rumours online is roughly quantitative, including descriptive studies of trace data [7–9], theoretical research on network factors [10, 11], and prescriptive studies that experiment with machine learning methods to classify rumours as true or false [12, 13]. Kwon et al. [8] include a descriptive analysis of temporal characteristics; false rumours on Twitter have more spikes than true rumours. Quantitative understanding of rumours focuses on how people participated in the rumour discussions and how the rumour developed over time. For instance, it could lead to the extraction of patterns in the text content, or different user roles. Rumour analysis has gained from studies in the related fields of meme-tracking [14], diffusion [15, 16] and virality [17, 18] in social networks, measuring the influence in networks and information credibility estimation.

Yet few studies provide significant insight into *how* and *why* rumours spread, and classification research has been limited to distinguishing between true and false information. Current studies work like outlier detection of a specific database. Hence, they learn a local model that is specific to a social media, not applicable to another platform, and they speculate that a rumour is a negative message, like ‘spam’, which need to be rejected from the platform. One theory is nevertheless interesting in spreading rumor in a community [2]. They argue that transmission evolves in three steps: levelling, sharpening and assimilation. First step is deleting details, second step is keeping the main details, assimilation is transmission with noise. We can take advantage of social network datasets to test such theory. Taking the automatic content analysis and data mining processing of a message [19–21], we are interested in exploring the following research questions, summarised below:

Q1: Which features are relevant?
Q2: Can we model a rumourous event as a multi-spike event?
Q3: How is a rumourous text different from a non-rumourous text?
Q4: Can we observe levelling-sharpening-assimilation in datasets?

In our article, part 1 is dedicated to an extensive review of literature of 80 papers on rumours. Among them, 58, written after 2010, were about rumour studies, revealing recent interest in rumour/credibility/misinformation issues, and specifically with social media platforms. We made a synthesis of principal features used to describe rumours in these quantitative approaches. Feature selection is a key question in quantitative and modelling investigation. Part 2 presents the datasets we used for spread and content analysis. We used not only ad-hoc corpora for our studies, but also external databases, such as hoaxes/disinformation repositories and language corpora. Part 3 presents our modelling approach for rumour spreading and a comparison with a standard approach such as epidemiological models. Finally, part 4 shows a comparison of rumour corpora and event corpora with n-gram and skip-gram studies.

## Material and methods

### Related studies

#### Rumour theory

In psychology and sociology [1, 2, 22, 23] were first attempts to study rumor and showing increase errors across the retellings. Rumours can be hoaxes, jokes, little stories or information leaks [24–26]. But it can be also early reports during breaking news lacking enough support or evidence. If we look at the classification proposed by [27], we observe seven categories of rumours: computer virus alerts, superstitious chains, solidarity chains, petitions, hoaxes, urban legends, fun stories and funny photos/pictures. But [28] imagined another classification with nine topics: urban legends, commercial disinformation, political attacks, commercial offer attacks, false commercial offers, financial disinformation, defamation, loss of credibility operations and panic alert to induce terror. Often a rumour is dedicated to disturb VIPs [6]. Recently, others [29] have suggested that rumours are a communication strategy similar to speech acts [30, 31].

#### Rumour detection

**Fig 1:**
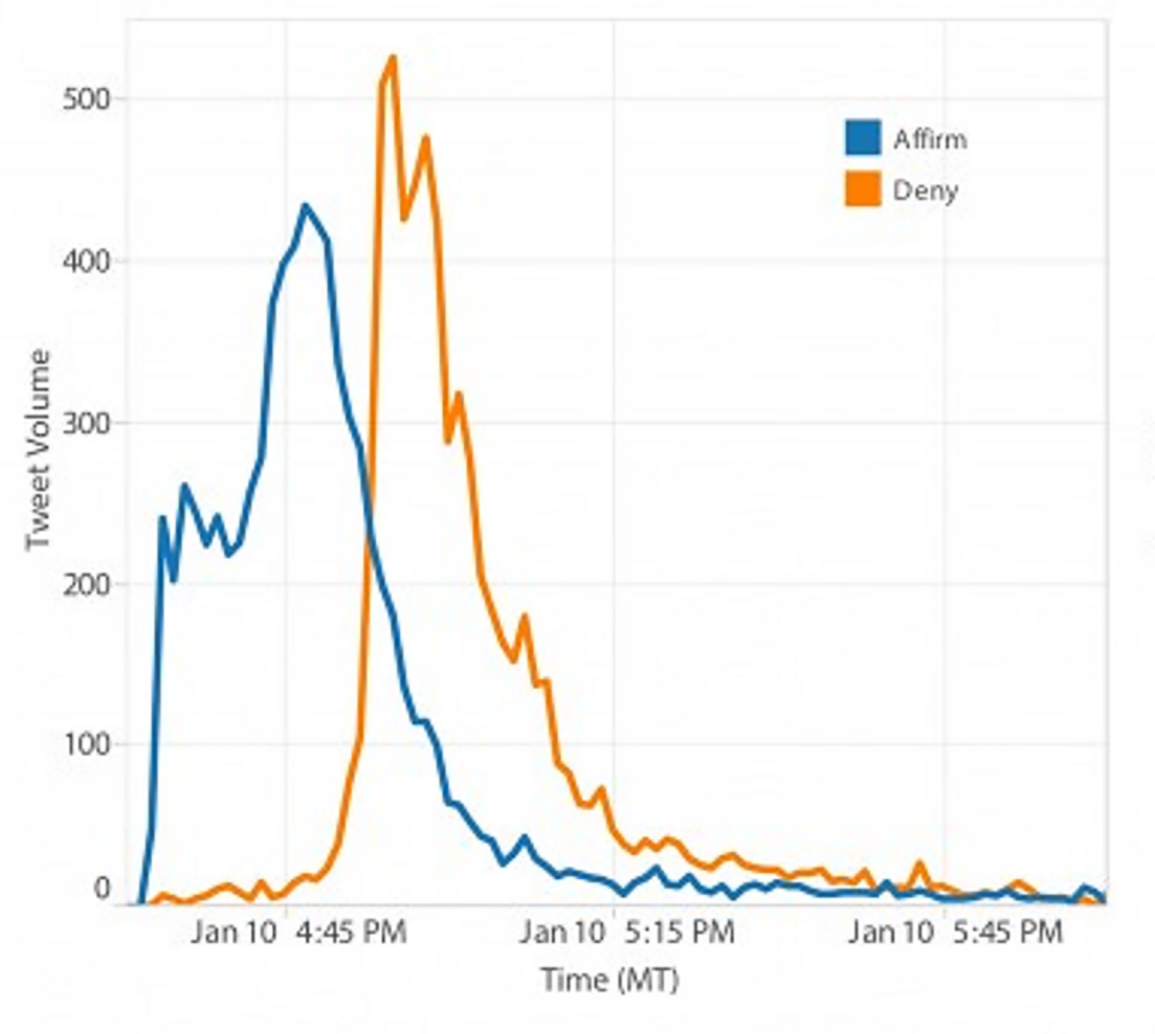
Propagation and denial of Westjet Hijacking rumor (tweets volume per minute, affirms versus denials)[40].

Recently, more computing studies have investigated the emergence of rumours, but they stay at the level of a specific rumour, as in Fig 1 [32–39].

Contrary to these studies, our goal is to analyse any kind of rumour and a corpus of rumours. Some systems claim to detect rumours but they are based on the similarity between an unknown message (i.e. email) and a well-known database of hoaxes or rumours [41–43]; other kinds of systems are more of a surveillance system for interesting message detection from the Internet (that are possibly rumours), and in this sense, they are more like an approximate recommendation system [44].

Formulation of the problem:

Microblog data can be modelled as a set of events = {*E*_*i*_}, and each event *E*_*i*_ consists of relevant microblogs for which we can associate a value for being or not being a rumour {*m*_*ij*_, *y*_*i*_}. An event *E*_*i*_ can be described by a set of *k* features from *l* different categories {*F*_*kl*_}. Hence, each message *m*_*ij*_ can be described by some values of these features. The most difficult case is to discover, in an unsupervised way, the value *y*_*i*_ for any message. In some cases we can know this value for a reduced amount of data from which we can learn a model (i.e. a profile), in a supervised way, and to detect similar messages.

[45] makes a good survey in the field of rumor detection. Most of the existing research uses common supervised learning approaches such as a decision tree, random forest, Bayes networks and a support vector machine (SVM). [46] imagined of first rumour detection system for the Chinese language and the Weibo social network. Weibo has a service for collecting rumour microblogs [47]. Qazvinian et al. [13] used a tagged corpus of 10,000 tweets of about five rumours, five categories of features (1-grams, 2-grams, Part-of-speech, hashtags, URLs) to classify rumours using the log-likelihood approach with good results (95% of accuracy) but they cannot apply their method to new, incoming, emergent rumours.

#### Rumour propagation

We can see rumour messages as a bag of documents, but also as a timeline with occurring messages. In that way, the formulation of the problem is a little different because it concerns the description of a discrete time series evolving over time [48].

Some previous work [49, 50] focuses on rumour propagation through the social network. They try to use graph theory to detect rumours and find the source of rumours. Virality is a major concept in rumour propagation [51], using epidemiological models, and some current studies still try to improve the models [52]. Spiro et al. [9] also model the rate of posts over time in their exploration of rumouring during the Deepwater Horizon oil spill in 2011. [53] identified five kinds of rumour statements, coded posts accordingly, and presented a model of rumour progression with four stages characterised by different proportions of each statement type.

The website TwitterTrails [54, 55] is one of the rare tools that does not present only a database but also intelligent information exploration (timeline, propagators, negation, burst, originator, main actors) in 547 social media stories. [10] prove that minimising the spread of the misinformation (i.e. rumours) in social networks is an NP-hard problem and also provide a greedy approximate solution.

Kwon et al [8] promoted uses of both temporal features, structural features and linguistic features. Linguistic features are related to the most words used in messages and taken from a sentiment dictionary (4,500 words stem). Network features are properties about the largest connected component (LCC). Temporal features point out periodicity of rumour phenomenon and give importance to an external shock that may incur not one but multiple impacts over time; here, the main feature is periodicity of an external shock. Fang et al. [56] describe a quantitative analysis of tweets during the Ebola crisis, which reveals that lies, half-truths and rumours can spread just like true news. They used epidemiological models. Fang et al. [56], studying 10 rumours about the Ebola crisis in 2014, claim that rumours propagate like news but they encourage quantitative analytics to distinguish news from rumours.

Granovetter [57] explains with its seminal work about weak ties, that some nodes in social networks mediate between different communities. Acemoglu et al. [58] give importance to bridges in social networks to spread biased beliefs. Menczer [59], in a talk for a world-wide web conference, underlined the importance of misinformation detection and fact checking, with goods results from machine learning techniques. Social media and traditional media work together to spread misinformation. Structural, temporal, content, and user features can be used to detect astroturf and social bots.

### Rumour sources

#### Disinformation sources

We are focusing on digital data that may be grabbed from the Internet. Others sources allow free access to misinformation like the website Emergent [60]. It monitors and evaluates the propagation of a rumour that has recently received a lot of attention. A new web service, emergent.info, developed by journalist Craig Silverman, is using journalists to evaluate online claims and deem them as true/false/unverified. They track the number of shares a rumour has on Facebook, Twitter and Google+ and report the numbers along with links to articles that support or counter the rumour.

We identified at least seven websites containing curated databases and serve as a reference to inform and to provide reassurance about rumours and disinformation on the web. These databases contain not only rumours but also hoaxes and jokes that may propagate on the Internet. ‘Snopes’ is the biggest, but with ‘hoaxkiller’, it is impossible to know how many articles it contains because the interface requires query function by keywords (Table 1).

**Table 1:** Disinformation web open databases.

Source	Language	#articles
hoaxbuster	French	292
hoaxkiller	French	?
hoax-slayer	English	2435
debunkersdehoax	English	340
hoaxes.org	English	4635
sites.google.com/site/dehoaxwijzer	Flammish	147
snopes.com	English	7289

‘Hoaxkiller’, ‘hoax-slayer’ and ‘dehoaxwijzersite’ are databases that display a list of hoaxes to show hoaxes and frauds. ‘Debunkersdehoax’ is a website that helps to invalidate rumours and disinformation from nationalists. ‘Hoaxes.org’ is a website that explores disinformation throughout history. ‘Snopes’ covers urban legends, rumours on the Internet and email, and other doubtful stories. We made a crawler (robot in perl language) to collect automatically the content of each website.

The famous and open encyclopaedia, Wikipedia, gives 220 as the number of existing social networks on Internet. These social media play as web 2.0 platforms with thousands till millions of active users where information as rumours can propagate quickly and easily. Twitter is one of them, and probably the most famous microblogging platform where 500 million tweets are published each day and 600 million users are registered, with 117 million active accounts publishing at least one tweet per month. Such a social platform is an ideal dissemination ‘relais’ for rumours. Two API (application programming interface) allows any computing programme to query the twitter database. Twitter Search API can index more than tweets but only from the previous seven days. Twitter Streaming API can retrieve more messages, but no more than 1% of the content per day.

From the database cited in Table 1, we compiled a corpus of 1,612 rumours (DIS-corpus) and disinformation texts among with 1,459 in English and 153 in French (81,216 tokens; 6,499 words).

Part 2 presents information sources and datasets. Part 3 is related to propagation. Part 4 addresses issues about information patterns in messages. We used R as the computing framework for modelling [61].

### Text data collections: Social media corpora and reference corpora

From Table 1, it is possible to see a sample of texts that is more related to rumours and disinformation because texts from databases are classified with categories. Hence, we were able to grab 1,612 texts discussing rumours (1,010 texts) and disinformation (602 texts). The size of the texts is relatively small, such as the news. But it is quite difficult to automatically select lexical information (by one or two words) that is typical from a given text. So we have manually chosen four texts and built a lexical query with two or three words to grab tweets from the Twitter social network (S2 Appendix).

From data collected in an open-access web database, we made a manual query to grab tweets from Twitter [62], and we built eight corpora to compare with the rumour corpora (Table 2).

**Table 2:** Three groups of datasets: First, rumour corpora; second, random corpora; third, event corpora.

Corpus	Language	#tweets	size (kb)	#tokens	#words
Holland	French	371	82	7,592	1,586
Lemon	French	270	49	13,611	3,451
Pin	English	679	118	31,612	6,691
Swine	English	1024	159	54,056	10,511
Random1_Fr	French	1000	131	72,387	15,449
Random2_Fr	French	1000	131	90,998	19,596
Random3_En	English	1000	135	110,657	24,580
Random4_En	English	1000	135	130,113	28,757
Rihanna_Fr	French	543	131	149,102	30,431
Rihanna_En	English	1000	81	160,295	32,264
Euro2016_Fr	French	1000	131	166,929	31,807
Euro2016_En	English	1000	147	188,882	32,771

The first rumour, ‘Hollande rumour’, is about the French political leader François Hollande. The rumour started in 2002 in private parties and in editorial offices. According the rumour scenario, the president of France–at that time he was deputy of the Correze region and first secretary of the labour party–was the father of one of Anne Hidalgo’s children, at that time, the First Executive Assistant of the Paris governor. Wikipedia’s description of Anne Hidalgo highlights that she had two children from a previous relationship. A black hole of information is sufficient to excite the web. The following query induced the retrieval of data:

*(hollande AND hidalgo AND fils) lang*:*fr*

The ‘lemon rumour’ pointed out that a lemon could cure cancer, saying it exceeds the power of chemotherapy by 10,000. The origin of this rumour is a Reuters news article in 2003, ‘An Orange a Day May Keep Some Cancers Away’. The following query induced the retrieval of data:

*(citron AND cancer) -femme-campagne-musique-arabes-punk-branché-limonade-Kickstarter-gato-Crowdfunding-Baptême-court-CM-tittytuesday-morito-nestea-bracelet-aluminium-déodorant-déodorants-agrumes-puce-poils-tropic-art-astrologie-bouteille-crame-coude-photo-tartes-bronzage-olive-horoscope-bonbons-google-jeu-hypocrisie-rose-malboro-Ananas-Bronzage-quantitatif-Tropiques-Téflon lang*:*fr*

The ‘PIN rumour’ claimed that in New York, entering your personal identification number (PIN) backwards will automatically send a message to the police that you are in trouble and that they will respond to the machine. This rumour seems to have appeared in 2006. The reverse PIN system was first imagined in 1994 and patented in 1998 by Joseph Zingher but never adopted by the banking industry. The following query induced the retrieval of data:

*(pin AND atm AND police) lang*:*en*

‘Swine flu rumour’, related to the swine flu virus or officially called the H1N1 flu virus, mentioned that thousands of people were sent to the hospital during the soccer championship in 2009 in South Africa. The following query induced the retrieval of data:

*(“swine flu”AND “South Africa”) lang*:*en*

There are two kinds of reference corpora. The first group is random corpora made on Twitter with a stopword. We chose the first 1000 tweets for each operation, repeated two times and for both French and English. The second group is related to events, and we also collected data from Twitter in April 2016. First event is a concert in France in August 2016 by Rihanna. The second event is the UEFA Europe football championship in France in 2016. For both events, data was collected in French and English and we kept no more than 1000 tweets.

We used two reference corpora for comparison with common language and for each language (Table 3). FR-corpus is an open database that contains 500 literary works from the 18^th^ to 20^th^ century. It is a free sample of the Frantext online database containing 248 million words [63]. ER-corpus is a collection of news from the French local newspaper East-Republican (‘L’Est Républicain’) about 1999, 2002 and 2003 [64]. BNC-corpus is a collection of samples of written and spoken language of British English from the latter part of the 20^th^ century. The written part consists of extracts from regional and national newspapers, specialist periodicals and journals for all ages and interests, academic books and popular fiction, published and unpublished letters and memoranda, school and university essays, among many other kinds of text. The spoken part (10%) consists of orthographic transcriptions of unscripted informal conversations and spoken language collected in different contexts, ranging from formal business or government meetings to radio shows and phone-ins [65]. The COCA-corpus contains spoken texts, fiction, popular magazines, newspapers, and academic texts produced between 1990 and 2015. It is a free sample of the 520 million word original corpus [66].

**Table 3:** Content of reference corpora for French and English.

Corpus	Language	#Files	Storage (Mb)	#Words	#tokens
Est Républicain Newspaper (ER)	French	544	1,025	654.134	130,746,677
Frantext literary database (FR)	French	500	147	817.754	20,218,763
Contemporary American (COCA)	English	115	10	62.47	1,809,601
British National Corpus (BNC)	English	4.049	4,680	981.636	98,112,611

### Information propagation

#### Classical epidemiological models

In the Internet era, many studies about rumours have shown that that rumours disseminate as a disease contagion like a Poisson distribution. We tried to confirm this hypothesis.

We made two displays of propagation with our four rumours corpora. First, visualisation is obvious, and we can plot the occurrence of tweets as on a timeline in a histogram plot. We do not know the IP number of senders of a tweet but we can know if a tweet is a retweet, hence, if a tweet has been transmitted. More generally, we can study the natural language content of each tweet. Hence, the second visualisation concerns tweet grouping by similarity to explore their distribution over time.

**Fig 2:**
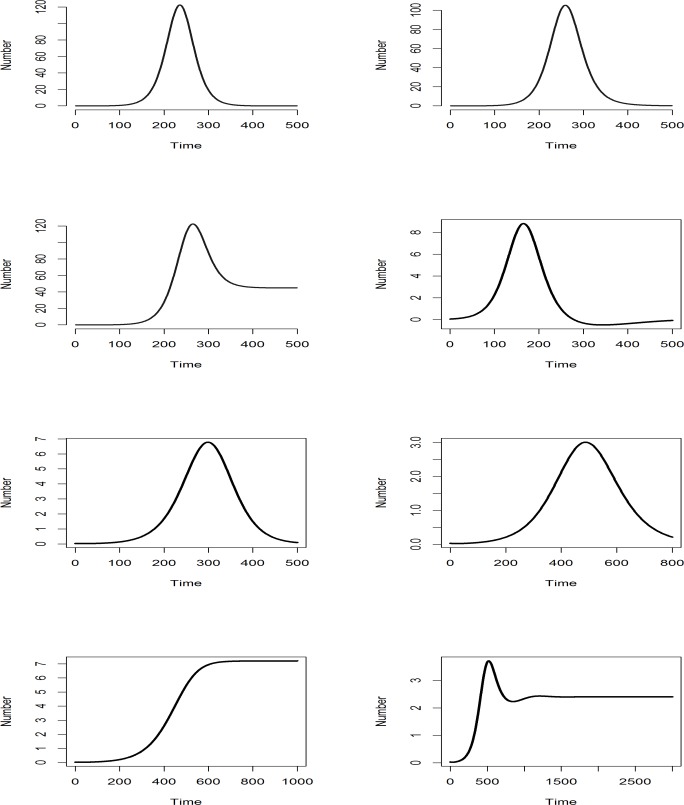
Displays of epidemiological model profiles (number of infected individuals over time). We can see at first line: SI model (left), SIR Model (right); at second line SIS model (left), SIRS model (right); at third line SEI model (left), SEIR model (right); at fourth line SEIS model (left), SEIRS model (right).

A rumour can be seen as a disease propagating over a population of sane individuals becoming infected over time. Several models are possible. Let be *S* the sensible population that is likely to be infected, *E* the population that is exposed, *I* the population that is infected and *R* the population that is cured. Eq (1) to Eq (18) summarise main models (Fig 2 shows the respective infected output for each model). The most simple is the SI (sensible-infected) model created by Hamer in 1906. In this model no individual can be cured. β Parameter is valued between 0 and 1. β∼*P*(*S*↔*I*)*P(*S*→*I*), where *P*(*S*↔*I*) is the probability that a sensible individual will be in contact with an infected individual, and *P*(*S*→*I*) is the probability that a sensible individual becomes infected if they are in contact.

(a) SI model
  $$\begin{array}{l} \frac{\beta SI}{N}\;new\;infected\;number\;per\;day\\ \frac{dS}{dt}=-\frac{\beta SI}{N}\\ \frac{dI}{dt}=\frac{\beta SI}{N} \end{array}$$ Eq (1)
(b) SIR model
  $$\begin{array}{l} \frac{dS}{dt}=-\frac{\beta SI}{N}\\ \frac{dI}{dt}=\frac{\beta SI}{N}-\gamma I\\ \frac{dR}{dt}=\gamma I \end{array}$$ Eq (2)
(c) SIS model
  $$\begin{array}{l} \frac{dS}{dt}=-\frac{\beta SI}{N}+\gamma I\\ \frac{dI}{dt}=\frac{\beta SI}{N}-\gamma I \end{array}$$ Eq (3)
(d) SIRS model
  $$\begin{array}{l} \frac{dS}{dt}=-\frac{\beta SI}{N}+fR\\ \frac{dI}{dt}=\frac{\beta SI}{N}-\gamma I\\ \frac{dR}{dt}=\gamma I-fR \end{array}$$ Eq (4)
(e) SEI model
  $$\begin{array}{l} \frac{dS}{dt}=-\frac{\beta SI}{N}\\ \frac{dE}{dt}=\frac{\beta SI}{N}-\varepsilon E\\ \frac{dI}{dt}=\varepsilon E \end{array}$$ Eq (5)
(f) SEIR model
  $$\begin{array}{l} \frac{dS}{dt}=-\frac{\beta SI}{N}\\ \frac{dE}{dt}=\frac{\beta SI}{N}-\varepsilon E\\ \frac{dI}{dt}=\varepsilon E-\gamma I\\ \frac{dR}{dt}=\gamma I \end{array}$$ Eq (6)
(g) SEIS model
  $$\begin{array}{l} \frac{dS}{dt}=-\frac{\beta SI}{N}+\gamma I\\ \frac{dE}{dt}=\frac{\beta SI}{N}-\varepsilon E\\ \frac{dI}{dt}=\varepsilon E-\gamma I \end{array}$$ Eq (7)
(h) SEIRS model
  $$\begin{array}{l} \frac{dS}{dt}=-\frac{\beta SI}{N}+fR\\ \frac{dE}{dt}=\frac{\beta SI}{N}-\varepsilon E\\ \frac{dI}{dt}=\varepsilon E-\gamma I\\ \frac{dR}{dt}=\gamma I-fR \end{array}$$ Eq (8)

#### Harmonic modelling

A harmonic oscillator is an ideal oscillator that evolves over time by a sinusoid, with a frequency independent of the systems properties, and the amplitude is constant. Oscillations can be damped, and the equation is hence written as follows:

$$\begin{array}{c} \frac{d^{2}s}{{dt}^{2}}+\frac{2}{\tau }\frac{ds}{dt}+\omega_{0}^{2}x(t)=0 \end{array}$$ Eq (9)

If $\omega_{0}>\frac{1}{\tau }$ state is sub-critical, solution is a damped oscillation with such pulsation:

$$\omega =2\pi f=\omega_{0}.\sqrt{1-\frac{1}{\tau^{2}\omega_{0}^{2}}}$$ Eq (10)

$$\begin{array}{c} s(t)=A.e^{-\frac{1}{\tau }}.cos(\omega t+\varphi_{0}) \end{array}$$ Eq (11)

where *A* is the amplitude, *f* is the frequency, *φ*_0_ the phase to origin, *ω* the pulsation, *τ* the relation time.

#### Models implementation

Epidemiological model displays were done with R with the basic plot function. Experimental implementation of harmonic modelling was done by fast Fourier transform using fft function and least-square in R using function nls (stats package) [61].

### Rumour lexical content

#### Frequent syntagmatic extraction

In this part we try to understand what kind of combinations can be typical of a rumour or a set of messages about a specific rumour.

We can set two main kinds of combinations. The first ones are lexical n-grams. A lexical n-gram is a sequence of n contiguous words separated by a blank. If n = 1, it is a simple word (as we can see in any dictionary entries for instance) if n>1, it is what it is named in linguistics ‘collocations’. Some collocations can be paradigmatic and then they are named ‘phrases’ (if they do not contain verbs, they are named ‘noun phrases’). The second kind of combination is a set of 1-gram separated by an n-gram not included in the combination. In case such a combination consists of two n-grams, it is named ‘co-occurrence’; in the cases where it is several n-grams, it is called a ‘frequent itemset’. We can also find the word ‘skipgram’, by analogy of n-gram.

#### Rare syntagmatic extraction

We tested the capacity of a rumour text to involve a non-standard combination of words. For such studies we used common languages corpora. The first experiment is an extraction of cleaned n-grams, and we checked presence/absence in reference corpora. The second experiment is a check of frequent skipgrams consisting of most frequent simple words.

In the first experiment we measured originality of a given corpus by the ratio *MW*_*c*_ of n-grams not included in a reference corpus by the number of total segments. We used 12 corpus among those four rumours corpus, but also randomly constituted corpora, and corpora based on recent real-world events in French and in English (in the present case: Rihanna concert in Europe in summer 2016, and UEFA Euro 2016). The measure *MW*_*c*_ is expressed as follows:

$$\begin{array}{c} {MW}_{c}=\frac{{NMW}_{c}(no)}{{NS}_{c}} \end{array}$$ Eq (12)

where *NMW*_*c*_ = *NMW*_*c*_ (no)+ *NMW*_*c*_ (yes) with *NMW*_*c*_ is the number of multiwords in the corpus c and *NMW*_*c*_ (no) is the number of multiwords not contained in a language reference corpus (for instance COCA-corpus for English).

#### Syntagmatic combination analysis

Finally, the next step after analyzing lists of features of 2 or 3 words is to measure the incidence of content with vector of words. For that, we cannot use the DIS-corpus because each rumour is unique and a set of ten or twenty words could not show similarity with other rumours. But if we take the Twitter rumours, we can observe how people talk about a rumour and compare the specificity of rumour discourse with ordinary messages.

We would like now get an overview of words importance in the rumorous content over time. Recall that (Allport, and Postman, 51) specifies a rumor mechanisms in three different mechanisms applicable in any situation. The first mechanism is a selection of main features (leveling, or loss of details). The second mechanism is sharpening refers to is an emphasis of some details during the transmission. Finally the last mechanism, assimilation refers to a distortion in the transmission of information. Linguistic assimilation usually consisted of inserting the words "is," "is as," "as," or "it's" or noise. Let suppose a rumor starts with nine details and ends with three, they would say that six were leveled and three were sharpened.

Our empirical studies is done in four steps:

- first step is lexical preprocessing of the dataset—splitting data into elementary words.
- second step is time preprocessing of the dataset—splitting dataset into 7 timestamps (getting enough data in each chunk at least 50 messages).
- third step is subset preprocessing of the dataset—splitting word features into three box according Zipf law saying that lexical distribution is always distributed into a small set of high frequency, medium frequency set words, and big set of low frequency.
- fourth step is computation of transitions.
- fifth step is plotting transitions.

We implemented the scripting in R platform, using regular expression for lexical splitting, ‘intersect’ function for calculation of transitions and GMisc’package ‘transitionplot’ for display of transitions.

Another angle to capture association is machine learning algorithms. Why, because machine learning algorithms use features, often within non-linear techniques indirectly taking into account combination of features. In summary, it captures correlation of features to make a good prediction without specifying association between features. We used four famous algorithms to make prediction: ‘Maxent’, ‘Random Forest’ (regression tree), ‘SVM’ and ‘SLDA’ (topic model). The first question that arises, due to sensitivity of algorithms to the feature space, is to define the dimensionality of the feature space. We can take the whole set of words (between 3,000 and 4,000 words) but it can be time consuming for some techniques or noise generation. We make a documents x terms matrix using different samples, i.e. the 10, 50, 100, 150, 200 and 300 most frequent words. We consider that rumorous messages starting by the same 70 characters (half of the message) are the same and we delete them for building the dataset. Hence the dataset consists of 1,678 messages containing all the four rumors messages, the pool of message to predict. We mixed this subset with 9,818 non-rumor messages. As training dataset we chose all the rumor subset and 2,000 non-rumor messages. As test dataset we take the 1,648 rumorous messages (17%) and 8,170 non-rumorous messages (83%). As baseline for comparison of techniques we consider the random assignment. A message can be assigned randomly as rumorous or non-rumorous. So the success rate is 50% percent of accuracy. Let suppose we classify all messages as non-rumorous we get 83% of accuracy but we lost all rumorous prediction because accuracy for rumorous will be 0%. Hence for each classification method we compute two indicators that are the global accuracy that we want enough high better than random for a stream of both rumorous and non-rumorous messages, and accuracy specific for rumorous messages that we expect also close to random score.

In the next experiment we keep the same matrix as before with 100 most frequent feature space but we change the document space. We make three submatrix: the first submatrix is 100% of the document space (1,618 rumorous messages), the second submatrix is the first 30% over time (498 rumorous messages), the last 30% over time (524 rumorous messages). Amount of non-rumorous messages in test set is always about 8,000 messages, and for the train set we keep the same amount than the rumorous set (about 500 or 2,000 messages).

#### Models implementation

The experimental implementation was done in R. The syntagmatic extraction is a function using regular expression analysis with gsub function (base package), multi-word extraction with ngram function (ngram package), and data cleaning using a stopwords list. Classification models were created using train_model function (RTextTools package) [61].

## Results

### Spreading modelling

**Fig 3:**
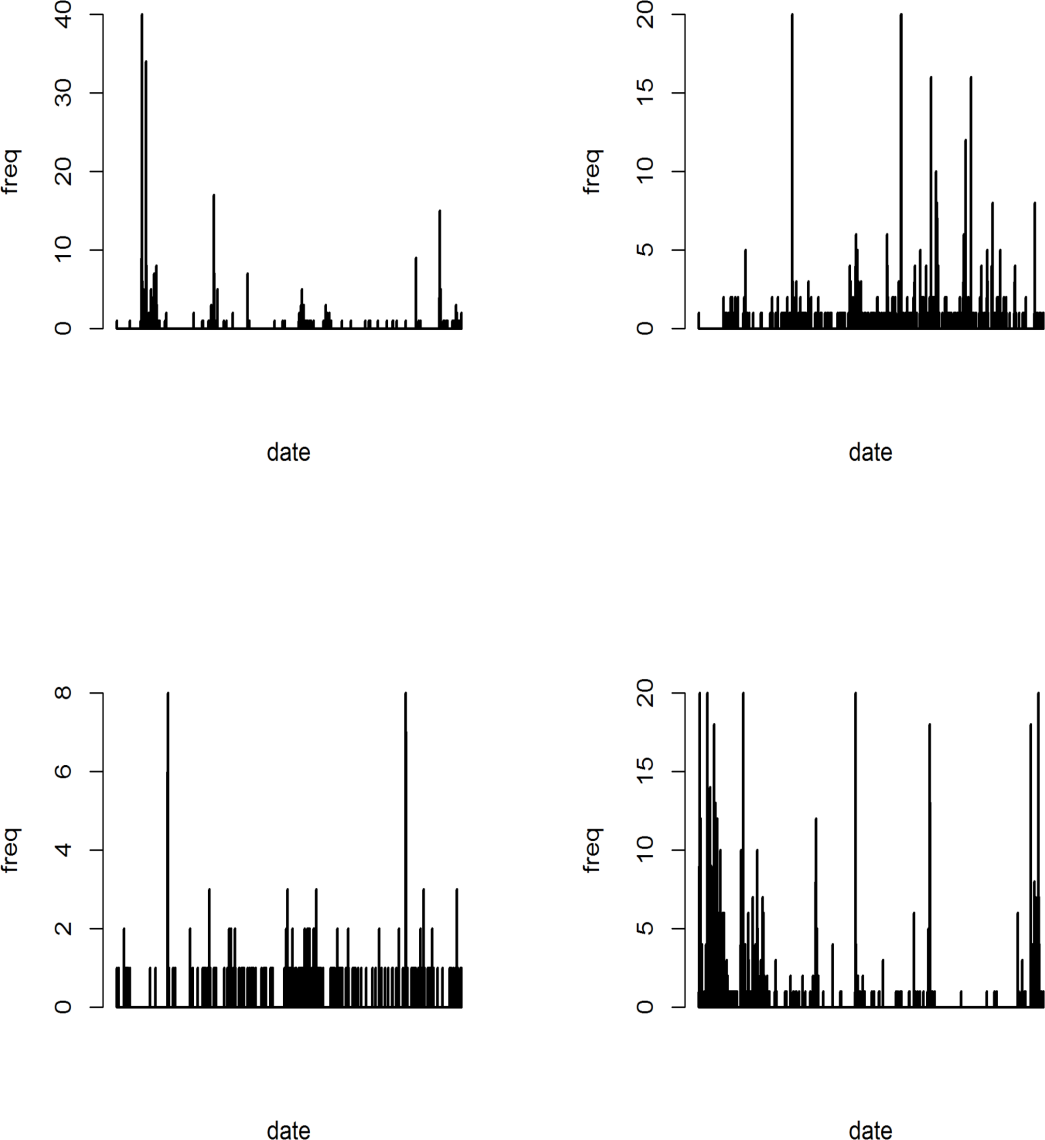
Displays of number of infected individuals over time for each epidemiological model (upper left: Hidalgo-corpus; upper right: PIN-corpus; bottom-left: Lemon-corpus; bottom-right: swine-corpus).

Fig 3 displays time distribution of tweets emission by users for each rumour. We can see that no plot really can fit with a 2-local maximum distribution, as shown on Fig 2.

**Fig 4:**
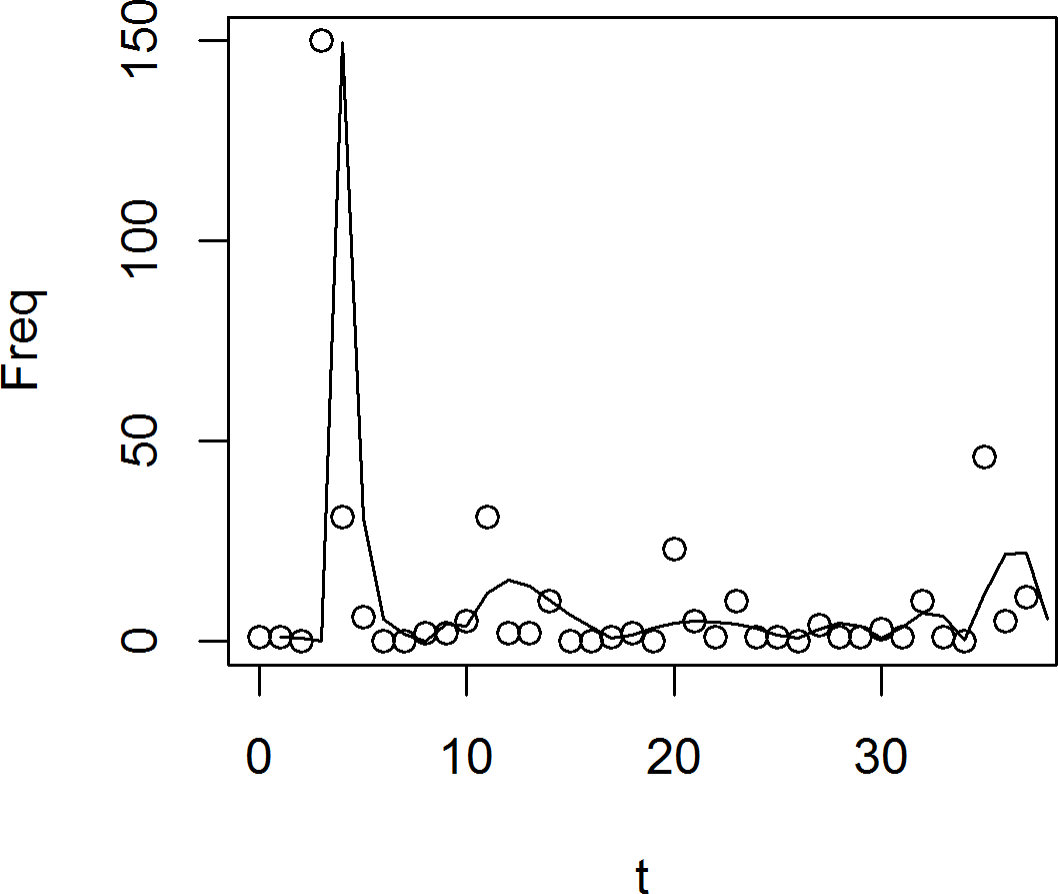
Fitting between a harmonic oscillator model and tweet distribution emission over time.

Fig 4 shows fitting of the Hidalgo-rumour corpus and the oscillator model with the setting: *A* = 10, *φ*_0_ = 15, *τ* = 23, *f* = 0.3.

**Fig 5:**
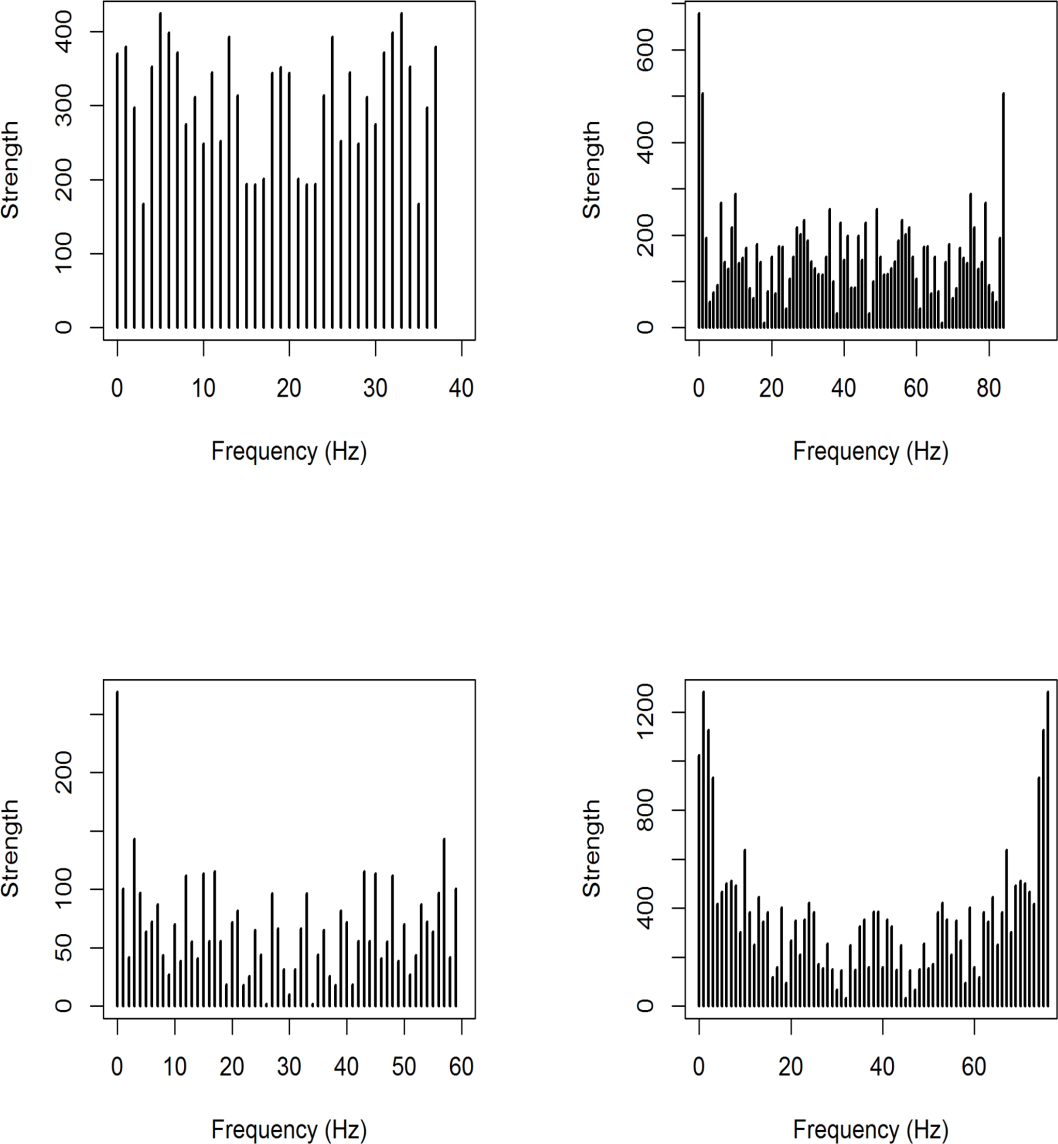
Displays of frequencies by fast Fourier transform for each corpus (upper left: Hidalgo-corpus; upper righ t: PIN-corpus; bottom-left: lemon-corpus; bottom-right: swine-corpus).

An advantage of the oscillator model is that it produces several local maxima (see Fig 5), whereas epidemiological models produce only one or two local maxima.

Fig 4 shows us a fit of Hidalgo-corpus with a damped oscillator model. It fits quite well, and better than any epidemiological model. But it seems that amplitude is not stable.

$$\begin{array}{c} s(t)=\sum_{i=1}^{n}A_{1i}.e^{-\frac{t}{A_{2i}}}.cos(A_{3i}t+A_{4i}) \end{array}$$ Eq (13)

$$A_{11}=48.9,\;A_{21}=8.8,\;A_{31}=0.36,\;A_{41}=14.5,$$

$$A_{21}=234.5,\;A_{22}=2.37,\;A_{23}=1.10,\;A_{24}=-0.11,$$

$$A_{31}=501.9,\;A_{32}=1.28,\;A_{33}=3.83,\;A_{34}=-20.8,$$

$$A_{41}=-0.0036,\;A_{42}=-4.00,\;A_{43}=51.0,\;A_{44}=-16.5$$

### Frequent syntagmatic extraction

Table 4 shows us a list of frequent n-grams for each corpus of rumours: Hidalgo-corpus, Lemon-corpus, Pin-corpus and swine-corpus. ‘Counting’ is the number of occurrences in terms of documents about cleaned n-grams. We cleaned n-grams by subtracting the prefix or suffix matching with stopwords. Processing is done in both languages.

**Table 4:** List of 30 most frequent words and noun phrases in rumours corpora (Holland, lemon, Pin, swine).

Hidalgo-corpus	frequency	Lemon-corpus	frequency	pin-corpus	frequency	swine-corpus	frequency
hollande	256	cancer	203	police	629	flu	807
caché	216	citron	200	reverse	626	south	801
hidalgo	184	contre	46	atm	624	swine	795
fils	161	ennemi	38	pin	622	africa	792
françois	128	plus	37	pin reverse	475	south africa	791
censure	123	contre cancer	37	will	289	swine flu	781
enfant	123	n°1	31	entering	259	cases	141
enfant caché	121	ennemi n°1	31	call	186	#swineflu	115
caché censure	120	ennemi n°1 cancer	30	+alert	166	h1n1	115
enfant caché censure	120	n°1 cancer	30	alert	166	news	114
françois hollande	119	citron ennemi	29	money	159	health	107
twitter	116	jus	27	entering your pin	155	world	100
hollande hidalgo	114	citron ennemi n°1	26	atm pin	138	cup	92
caché censure twitter	114	fois	25	atm will	131	flu south	91
censure twitter	114	puissant	25	reverse any atm	128	flu south africa	87
hidalgo enfant	111	fois plus	24	enter	112	world cup	87
hidalgo enfant caché	111	jus citron	23	call the police	108	swine flu south	84
hollande hidalgo enfant	109	santé	22	will not call	97	confirmed	81
fils caché	94	thé	22	alert the police	95	#h1n1	76
rumeurs	84	plus puissant	21	atm pin reverse	91	outbreak	66
non	82	#cancer	20	rumors	87	flu cases	65
compagne	81	cancer citron	20	contrary	86	swine flu cases	65
divorcée	81	0	19	rumors entering	86	death	63
compagne non	81	ovaire	19	popular	85	reported	55
compagne non divorcée	81	000 fois	19	thief	83	news24	55
non divorcée	81	000 fois plus	19	contrary popular	83	flu death	51
caché compagne	80	fois plus puissant	19	contrary popular rumors	82	africa swine	50
caché compagne non	80	guérit	16	popular rumors	82	africa swine flu	50
fils caché compagne	80	cancer ovaire	16	popular rumors entering	82	swine flu death	50

In Table 4 no information appears to make sense for a rumour in general. We mostly distinguish lexical patterns clearly related a given rumour like ‘flu death’, ‘h1n1’, ‘Africa swine’, ‘flu cases’ for swine corpus.

If we look at Table 4‘s top four lexical strings, we see that only simple words appear; it is a general observation that stopwords are more frequent than simple words, and simple words are more frequent that multi-words. Next we tried to extract the most frequent simple words over the 1,612 rumourous texts (1,459 in English, 153 in French). Table 5 shows the most frequent words in the database by decreasing order of occurrences or documents. If we set a threshold such as 10% of documents (146 in English, 15 in French) and if we consider the number of occurrences, we observe that only 20 simple words are significant. Among these words we can see only two words about a specific topic (cancer, Obama) and no word very typical for a rumourous alert. If we consider the number of documents, 160 words are relevant (64 in French, 96 in English). Most of words are very short (two or three characters). We cannot see any named entity in these lists (person’s name, organisation, product names). Many words seem to be tool words such as: *pro*, *ex*, *hey*, *side*, *app*, etc. Another big cluster of words are general verbs such as *go*, *use*, *eat*, *see*, etc. Some general meaning words seems recurrent too such as *men*, *one*, *day*, *king*, *war*, *ease*, etc. We cannot extract any global argumentative structure of a rumour that is redundant across a large set of documents.

**Table 5:** Common words for English in DIS-corpus sorted by decreasing frequency order (right by occurrences count, right by document count).

Word	Freq	Word	Freq	*french*				*english*					
obama	584	american	221	*word*	*freq*	*word*	*freq*	*word*	*freq*	*word*	*freq*	*word*	*freq*
people	437	back	183	an	152	elles	57	er	1393	pa	851	sc	471
know	419	told	183	al	142	autre	57	re	1383	nc	817	king	465
just	405	world	183	si	138	lors	57	ed	1350	rd	806	day	460
said	379	take	177	or	138	avoir	56	ing	1337	ill	790	dr	458
president	341	years	173	el	136	rien	56	st	1312	eve	765	ran	454
please	336	country	168	no	134	personne	56	hi	1281	one	762	side	450
plus	297	think	164	ans	132	main	56	nt	1274	ear	745	know	442
like	261	cancer	160	ca	127	car	55	ve	1238	go	671	ring	440
time	244	make	149	com	124	puis	55	al	1238	use	670	old	438
				lu	123	vers	53	ll	1216	ap	670	sin	436
				va	121	toute	52	de	1166	com	660	son	430
				ni	121	of	52	co	1152	ny	654	app	429
				air	118	fois	51	ma	1128	men	630	rat	429
				mme	117	pris	51	ca	1123	end	618	era	426
				and	111	grand	50	us	1121	ga	604	lt	421
				pu	100	met	50	ur	1105	ex	596	tim	420
				dr	100	parti	49	hat	1098	pro	583	car	419
				art	100	porte	48	ho	1073	man	581	ass	416
				plus	99	autres	47	el	1069	hey	581	ms	410
				tant	99	dire	47	la	1037	now	579	war	406
				don	91	prend	47	wa	1020	ain	579	get	402
				tout	89	cour	47	id	1013	ever	575	pen	396
				ali	89	donc	44	un	990	red	569	ease	394
				fait	88	loi	44	ad	960	ok	564	ten	390
				vie	82	quelqu	44	lo	941	per	526	cause	389
				cons	82	auto	44	em	928	ice	513	thing	383
				voir	81	peut	43	rt	896	thin	507	low	381
				jour	81	mal	41	sh	887	age	489	aid	375
				comme	79	nation	41	ate	875	act	488	people	373
				sent	78	vient	40	im	864	eat	481	inc	366
				part	76	quelque	40	mo	858	led	477	see	365
				eau	74	nouvel	40	ted	853	ally	474	way	360

Table 6 represents another view of word frequency in the text database. It points out the distribution of lexical units (1-grams) over each database (French, English). We kept only words occurring in more than 10% of the documents, and we are displaying the list of words by decreasing order of coverage per cent. More French words are involved because 10% of a small sample covers only 15 documents. For English documents only three words cover more than 25% of the corpus: *one*, *people*, *know*. These words are not informative about a rumour’s general representation. We can also find prepositions or adverbs such as *like*, *now*, *us*. For French, 17 words cover 25% of documents, and among those, only two words are semantically significant–*France*, *pays*–but very general in any case. Other significant words are logical and argumentative such as: *si*, *donc*; but they still have a very global meaning for a consequence or condition. Other less frequent words deal with different topics such as people and domestic policy. An interesting fact is that the word *true* is often used in a message claiming a falsehood.

**Table 6:** Common words for DIS-corpora (sorted by reverse frequency order).

*french*						*english*		
	doc	cov		doc	cov		doc	cov
plus	92	60.130719	mois	24	15.686275	one	439	30.089102
comme	75	49.019608	jusqu	24	15.686275	people	376	25.771076
si	74	48.366013	jours	24	15.686275	know	341	23.372173
fait	61	39.869281	islam	24	15.686275	please	302	20.699109
tous	59	38.562092	chaque	24	15.686275	said	298	20.424949
tout	58	37.908497	nombre	23	15.032680	now	277	18.985607
france	55	35.947712	gouvernement	23	15.032680	get	272	18.642906
faire	54	35.294118	vie	22	14.379085	new	267	18.300206
bien	54	35.294118	pourquoi	22	14.379085	time	266	18.231666
avoir	45	29.411765	paris	22	14.379085	like	258	17.683345
autres	45	29.411765	gens	22	14.379085	don	243	16.655243
donc	42	27.450980	pendant	21	13.725490	true	239	16.381083
fois	41	26.797386	loi	21	13.725490	obama	224	15.352981
entre	41	26.797386	hui	21	13.725490	us	215	14.736121
non	37	24.183007	elles	21	13.725490	president	210	14.393420
pays	36	23.529412	droit	21	13.725490	take	205	14.050720
ainsi	36	23.529412	ceux	21	13.725490	make	205	14.050720
encore	34	22.222222	aujourd	21	13.725490	also	199	13.639479
depuis	34	22.222222	femmes	20	13.071895	back	197	13.502399
alors	34	22.222222	dit	20	13.071895	many	195	13.365319
peut	33	21.568627	autre	20	13.071895	going	192	13.159698
monde	33	21.568627	toujours	19	12.418301	go	191	13.091158
deux	33	21.568627	seulement	19	12.418301	see	190	13.022618
rien	32	20.915033	partie	19	12.418301	two	189	12.954078
personnes	32	20.915033	parce	19	12.418301	even	185	12.679918
information	32	20.915033	musulmane	19	12.418301	way	183	12.542838
avant	32	20.915033	grande	19	12.418301	first	177	12.131597
aussi	32	20.915033	euros	19	12.418301	found	176	12.063057
ans	32	20.915033	etat	19	12.418301	years	175	11.994517
temps	31	20.261438	demande	19	12.418301	told	175	11.994517
quelques	31	20.261438	certains	19	12.418301	may	175	11.994517
toutes	30	19.607843	aucune	19	12.418301	think	167	11.446196
moins	29	18.954248	attention	19	12.418301	friends	166	11.377656
enfants	29	18.954248	vers	18	11.764706	well	162	11.103496
car	29	18.954248	trop	18	11.764706	everyone	162	11.103496
vient	28	18.300654	pourtant	18	11.764706	around	158	10.829335
sous	28	18.300654	plusieurs	18	11.764706	man	157	10.760795
nouvelle	28	18.300654	mieux	18	11.764706	day	157	10.760795
dont	28	18.300654	suite	17	11.111111	never	155	10.623715
contre	28	18.300654	ministre	17	11.111111	want	150	10.281014
jamais	27	17.647059	faites	17	11.111111	pass	150	10.281014
afin	27	17.647059	etc	17	11.111111	last	150	10.281014
toute	26	16.993464	dernier	17	11.111111	world	146	10.006854
quand	26	16.993464	savoir	16	10.457516	called	146	10.006854
musulmans	26	16.993464	quoi	16	10.457516	every	145	9.938314
effet	26	16.993464	message	16	10.457516	use	143	9.801234
dire	26	16.993464	islamique	16	10.457516	read	142	9.732694
voir	25	16.339869	comment	16	10.457516	really	141	9.664154
selon	25	16.339869	bonne	16	10.457516	right	140	9.595613
personne	25	16.339869	aucun	16	10.457516	news	140	9.595613
grand	25	16.339869	article	16	10.457516	made	140	9.595613
cas	25	16.339869				come	140	9.595613
va	24	15.686275				say	138	9.458533
peu	24	15.686275				american	138	9.458533

We would like now get an overview of words importance in the rumorous content over time.

**Fig 6:**
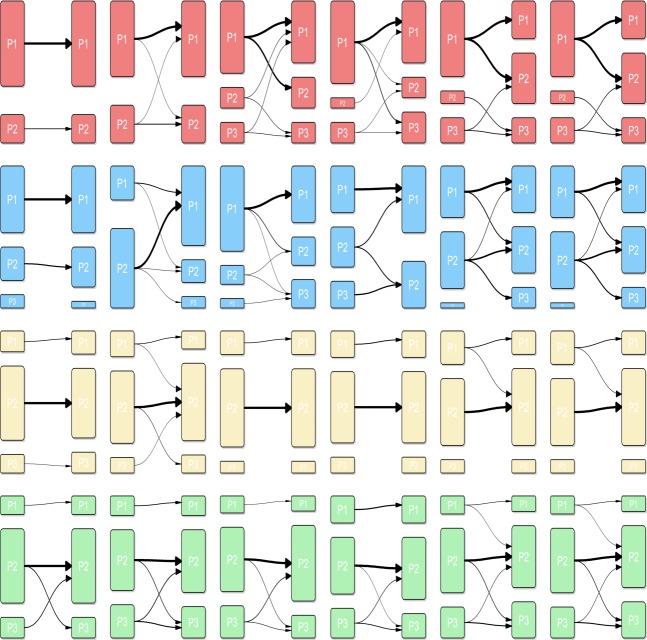
Lexical transfer from a period of time to the next for each rumorous datasets. Each line means a rumorous dataset (in red lemon, in blue: hidalgo, in yellow: pin, in green: swine-flu). Horizontal axis is the timeline. Each dataset is divided into 7 boxplot, generating 6 transitions. Each boxplot contains three frequency boxes. Top frequency box represent high frequency (around 10 words), the bottom frequency box represent 60% of lowest frequency words. The medium frequency box contain the remaining words.

Rumorous datasets were initiated before creation of twitter platform except for ‘swine flu’ that emerged in 2009. About ‘lemon’, ‘hidalgo’, and ‘pin’ we can not observe the levelling step. About ‘swine flu’ we do not observe any loss of lexical information at beginning of the rumour propagation (see Fig 6).

Sharpening in a transition point of view can be seen as frequent words that can become more frequent. Assimilation can be seen as noise words that come in and out. Our transition diagram can differentiate growing in frequency details (transfer from low and medium boxes to high frequency box)–i.e. sharpening—and capturing noise (transfer from low to medium boxes)–i.e. assimilation. We could see a sharpening in Fig 6 if the size of the arrow in our diagram increases, but it is not the case in any rumor.

On Fig 6 we can observe streams of words come in and out from low frequency box to medium frequency box in all rumorous transmission.

### Rare syntagmatic extraction

Table 7. Shows the results about measure *MW*_*c*_.

**Table 7:** *MW*_*c*_ measure for each tweets corpus.

	random1	random2	random3	random4
*MW*_*c*_	0.366500829	0.341423948	0.235514019	0.265442404
	H	Lemon	Pin	swine
*MW*_*c*_	0.7090301	0.585551331	0.697626419	0.641923436
	RiFr	RiEn	EuroFr	EuroEn
*MW*_*c*_	0.519650655	0.75060241	0.736717828	0.798293251

The second experiment is based on simple words shown in Tables 5 and 6 from which we made a file of 144 simple English words; we computed all combinations between two words (2-skipgrams) and three words (3-skipgrams). Hence, we checked the presence or absence of each skipgram in the corpora of common language in English (COCA-corpus).

In Table 8 we see that only five 3-skipgrams are not inside the common language corpus:

- obama please thing
- alert obama sh
- number obama please
- alert info obama
- don obama please

**Table 8:** Skipgrams of DIS-corpora included or not included in the COCA corpus.

	yes	no	*total*
**2-skipgrams**	10296	0	*10296*
**3-skipgrams**	487339	5	*487344*
*total*			*497640*

Specificity of these combinations is clearly related to the Obama name and cannot provide information about rumour structure in general.

### Syntagmatic combination analysis

On Fig 6 we can see different groups of similar messages for Hidalgo-corpus over time. At the beginning are two distinct groups of messages in bright blue and red, and at the end, a cluster in green. This figure shows us that during a flow of messages for a specific rumour, groups of similar messages can emerge in the same time window.

**Fig 7:**
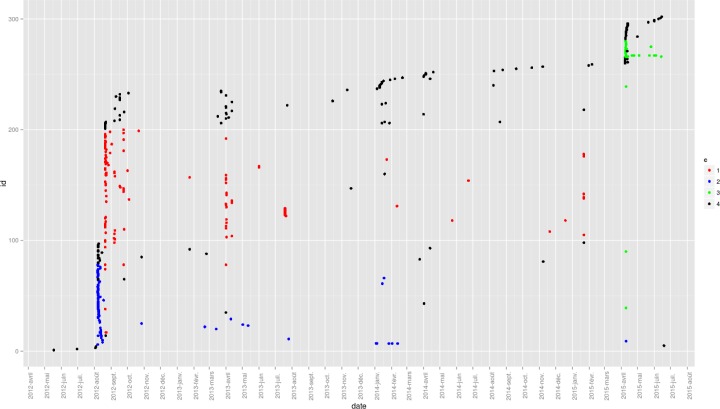
Clustering of messages according similarity of message for Hollande-corpus.

Fig 7 shows that bursts of similar messages occur over time, and leads us to think that indeed the content of rumour discourse is not heterogeneous.

We can suppose that a rumour discourse consists of local grammar and typical vocabulary in Twitter but also in the primitive short text. We plotted a timeline occurrence of rumours sorted (y-axis) by message similarity.

Another angle to capture association is machine learning algorithms that use features, often within non-linear techniques taking into account combination of indirectly correlated features.

**Fig 8:**
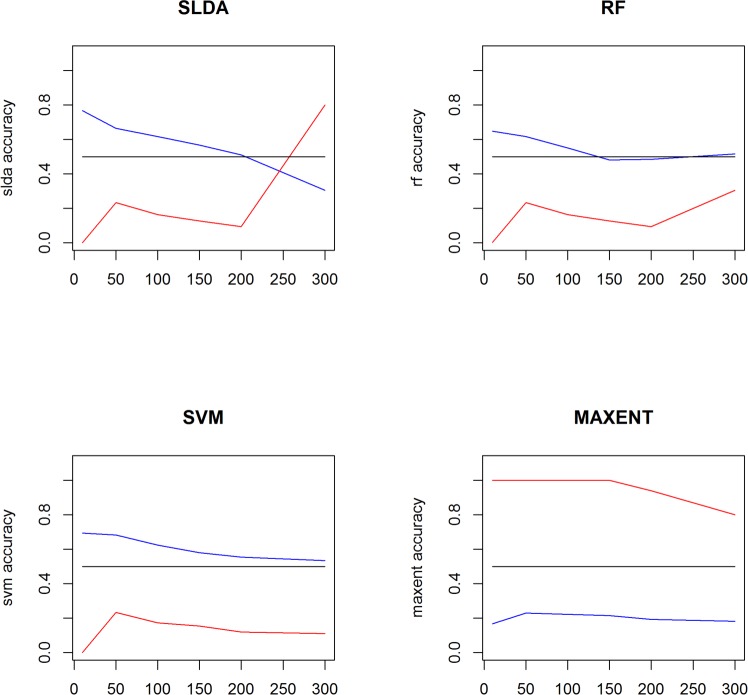
Classification performance (global accuracy rumorous/non-rumorous in blue; rumorous accuracy in red using following techniques: ‘SLDA’ (top left), ‘Random Forest’ (top right), ‘SVM (bottom left), ‘MAXENT’ (bottom right).

Fig 8 shows four plot for each classification methods. On each plot we have three curves: random (in black), rumorous accuracy (in red), global accuracy (in blue). We see that scores are not so good for a small amount of features (less than 50,) and scores degrade when they are more than 200 features. So we decide to keep the solution of 100 features.

**Fig 9:**
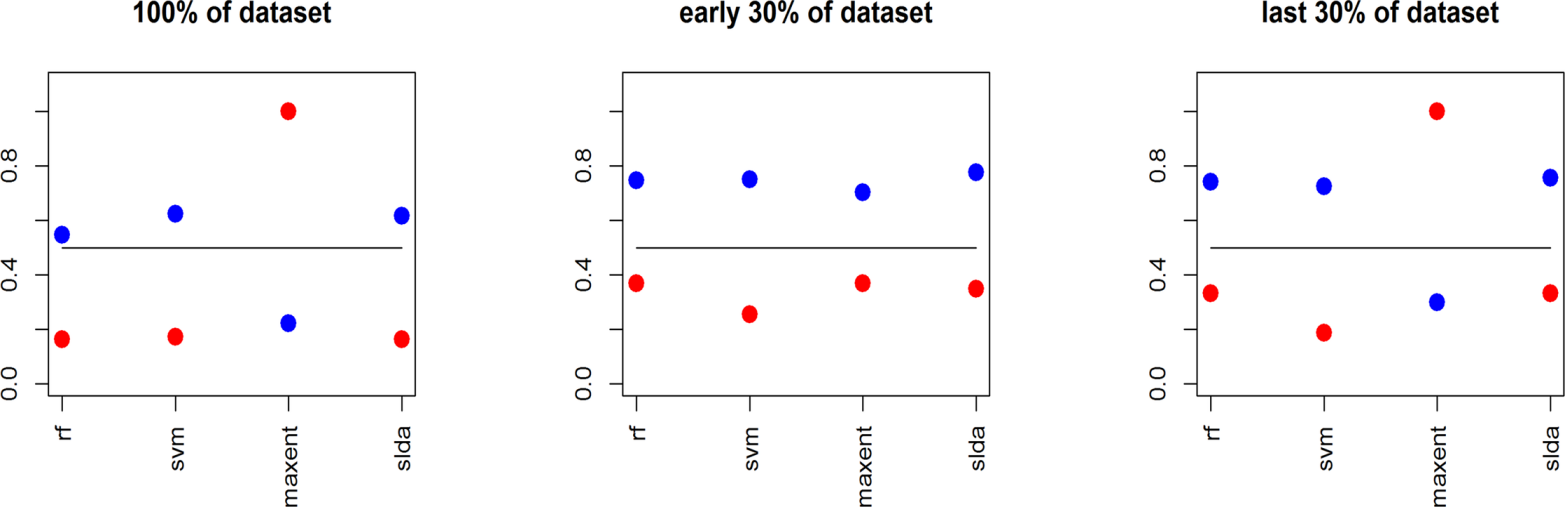
Classification techniques (Rf for ‘Random Forest’, ‘SVM’, ‘Maxent’, ‘SLDA’) applied on three samples: Whole rumorous dataset (left), the 30% first rumorous dataset in the range time (middle), the last 30% rumorous dataset in the range time (right). In blue the global accuracy (rumorous+non-rumorous), in red the rumorous accuracy (only rumorous), in black the random baseline.

Fig 9 shows the results. We can observe that the behaviour of predication is almost the same for Random Forest, SVM and SLDA and we see that there is a change between the overall dataset prediction behavior and the first 30% dataset, and the overall dataset keep the same behaviour as the 30% last dataset but with a degradation of performance in prediction.

It means an impact of the lexical composition over time that changed. Maxent seems to have a bad behaviori with low score of prediction. If we filter the number of prediction with more than 60% of certainty, we get only about 3,727 values, when other methods have about 9,500 values. When using the whole set of features (3,336, instead of 100 most frequent), the amount of values with high confidence raises to 7,351 but we still get only 9,2% for accuracy about the rumorous set when other methods get more than 33%. Maxent seems to work better with a highest dimensional space, but keeping a lower performance.

## Discussion

Our results show the complexity of rumour description and tracking in its diverse facets. Rumour analysis, being a psycho-social phenomenon, has regained interest because of social media platforms that relay news efficiently and widely, as well as events and information about important persons or organisations. Relevant studies have proven that the integration of specific features for automatic detection gives interesting results for case studies. Globally, there is no comparison of the difference between news and rumours. Furthermore, relevant features involved in models reveal that some misinformation lacks specific features or have more specific features, but each social media space can generate its own properties and because of this, rumours can spread with a combination of features that are not found in existing platforms (like Weibo or Wikipedia). Indeed we observed 53 features involved in models, but the combination of these features is high and it is not realistic to imagine a unique set of features to anticipate the shape of a rumour in a given digital context. Globally detecting rumours can be implemented locally in the context in which it is spread for a specific category of users. Can we imagine a connected world without rumours? Language evolves in any social world, and a rumour is in itself a marker of the language at a rhetorical level. So rumours can evolve in the same way that language evolves. For instance, a series of hashtags in a microblog can be a new kind of message, but in the same way a new kind of rumour construction. A rumour lifecycle evolves naturally like a scientific hypothesis, requiring confirmation or denial by other publications; in this sense, the majority of people socially accept this rhetorical process.

## Conclusion

To complete rumour and disinformation studies widely explored by qualitative means, we decided to investigate quantitative issues across any data sources. We studied several rumour datasets leading to a disinformation corpus of 1,612 rumourous texts (in French and English) from which we chose four rumours (French Hidalgo politician, lemon and cancer, ATM PIN code and swine flu in South Africa). We manually built two or three keyword queries to get tweets data about these four corpora. About the propagation of each rumour over time, we highlighted different profiles that may be either epidemiological-based but multi-harmonic-based. Focusing on the disinformation corpus we found that the intrinsic lexical content of rumours themselves has no specific content in term of lexical patterns when we compared them with reference corpora for the English or French common language, or to the corpora of event-based tweets. We tried also to highlight some previous theory of rumor argueing a transmission in three steps: levelling-sharpening-assimiliation. Taken this as a basis, we consider social network data as an empirical framework to provide data for validation of such theory. We can only confirm the assimilation part; we guess that levelling and sharpening occur enough early in dissemination and we do not observed it under the scope of 4 given rumors. So we distinguish two properties of rumors, largely disseminated in natural language (as a speech act) whereby they seem to have lexically no specific genre, and have a propagation with a certain resilience and assimilation process.

## Supporting information

S1 Appendix (DOCX)

S2 Appendix (DOCX)

## References

[1] Stern L. W. (1902). Zur Psychologie der Aussage: Experimentelle Untersuchungen über Erinnerungstreue. Zeitschrift für die gesamte Strafrechtswissenschaft, 22(2/3), 315–370.

[2] Allport G. W. and Postman L. The psychology of rumor. New York: Henry Holt, 1947, pp. 247 10.1111/j.2164-0947.1945.tb00216.x 21012248

[3] Metaxas P.T. 2010 Web spam, social propaganda and the evolution of search engine rankings. *Web Information Systems and Technologies*, 45, 170–182.

[4] Knapp R.H. 1944 A psychology of a rumor, Public Opinion Quarterly, 22–37.

[5] Morin E. (ed.), La rumeur d’Orléans (Translated in English: Rumour in Orleans), Paris, Seuil, coll « L’histoire immédiate », 1969

[6] Gaildraud L., Samier H., Bruneau J.M 2009. The generation of a rumour: from emergence to percolation, European Symposium of Competitive Intelligence (ECIS), Stockholm, Sweden, 11 th. & 12 th. JUNE 2009.

[7] Friggeri A., Adamic L. A., Eckles D., Cheng, J. 2014. Rumor Cascades. in Proc. 8th Int. AAAI Conference on Weblogs and Social Media (ICWSM).

[8] Kwon, S., Cha, K., Jung, W.C., Wang, Y. 2013. Prominent Features of Rumor Propagation in Online Social Media. Data Mining (ICDM), 2013 IEEE 13th International Conference Dec. 2013.

[9] Spiro E.S., Jeannette Sutton, Matt Greczek, Sean Fitzhugh, Nicole Pierski, Carter T. Butts. 2012. Rumoring During Extreme Events: A Case Study of Deepwater Horizon 2010. In Proceedings of the ACM Web Science 2012 Conference (WebSci12), 275–283. http://doi.org/10.1145/2380718.2380754

[10] Budak C., Agrawal D., El Abbadi A. 2011 Limiting the spread of misinformation in social networks In Proc. of WWW 2011, ACM, 665–674.

[11] Chierichetti F., Lattanzi S., Panconesi A. 2009 Rumor spreading in social networks In 36th Intl. Colloquium on Automata, Languages and Programming (ICALP), pp. 375–386. Springer.

[12] Castillo C., M. Mendoza, B. Poblete. 2011. Information credibility on twitter. In Proc. International Conference on World Wide Web, 28-Mar 01-Apr, Bangalore, India, 675–684

[13] Qazvinian Vahed, Emily Rosengren, Dragomir R. Radev, Qiaozhu Mei. 2011. Rumor has it: identifying misinformation in microblogs. In Proceedings of the Conference on Empirical Methods in Natural Language Processing (EMNLP '11). Association for Computational Linguistics, Stroudsburg, PA, USA, 1589–1599.

[14] Leskovec J, L. Backstrom, J. Kleinberg. 2009. Meme-tracking and the dynamics of the news cycle. In Proceedings of the 15th ACM SIGKDD international conference on Knowledge discovery and data mining, 497–506. ACM.

[15] Kostka J, Y. A. Oswald, R. Wattenhofer. 2008. Word of Mouth: Rumor Dissemination in Social Networks Structural Information and Communication Complexity. In A. A. Shvartsman and P. Felber (eds.). Structural Information and Communication Complexity, volume 5058 of Lecture Notes in Computer Science, chapter 16, 185–196.

[16] Kurihara S. The Multi agent based Information Diffusion Model for False Rumor Diffusion Analysis, WWW ‘14 Companion, 7–11 April 2014, Seoul, Korea.

[17] Serrano Emilio, Carlos Ángel Iglesias, Mercedes Garijo. A Novel Agent-Based Rumor Spreading Model in Twitter, Proceedings of the 24th International Conference on World Wide Web, 18–22 May 2015, Florence, Italy.

[18] Del Vicario M., Alessandro Bessi, Fabiana Zollo, Fabio Petroni, Antonio Scala, Guido Caldarelli H. Eugene Stanley, Walter Quattrociocchi. 2016 The spreading of misinformation online *PNAS* 113 (3), 554–559; published ahead of print 4 January, 2016, doi: 10.1073/pnas.1517441113 26729863 10.1073/pnas.1517441113 PMC4725489

[19] Charniak E. 1996 Statistical language learning. MIT press, Boston.

[20] Manning C.Schutze D., H. 1999. Foundations of statistical natural language processing. MIT press Cambridge, MA: 5 1999.

[21] Maimon O., Rokach L. 2005 Data mining and knowledge discovery handbook, Springer US, Editors Oded Maimon and Lior Rokach, ISBN 9780387098227.

[22] Kirkpatrick C.(1932). A tentative study in experimental social psychology. American Journal of Sociology, 38, 194–206.

[23] Bartlett F. C. (1932). Remembering: A study in experimental and social psychology. Cambridge England: Cambridge University Press

[24] Rosnow R.L Inside rumor: A personal journey. *American Psychologist*, 46(5), 484, 1991.

[25] Froissart P. 2004 Des théories sur la rumeur: pour quoi faire? (Theories about rumors: for which purpose?) *Les cahiers du GRÉDAM*. Paris: Université de Paris III, 2004.

[26] Lewandowsky S., Ecker U. K., Seifert C. M., Schwarz N., Cook J. 2012 Misinformation and its correction continued influence and successful debiasing. *Psychological Science in the Public Interest*, 13(3), 106–131. doi: 10.1177/1529100612451018 26173286 10.1177/1529100612451018

[27] Campion-Vincent V., Renard J-B. 2005 De source sûre: Nouvelles rumeurs d'aujourd'hui (In: reliable sources: new recent rumors), Payot, Paris.

[28] Heiderich D. 2004 Rumeur sur internet: Comprendre, anticiper et gérer les cybercrises (Rumor on internet: understand, anticipate and manage cybercrises). Village Mondial, Pearson Education France, Paris. ISBN 2-7440-6088-7.

[29] Bernardi D., Cheong P. H., Lundry C., Ruston S. W. 2012 Narrative Landscapes: Rumors, Islamist Extremism, and the Struggle for Strategic Influence. Rutgers University Press, New Jersey.

[30] Searle J. R. 1985 Expression and meaning: Studies in the theory of speech acts. Cambridge, University Press.

[31] Vosoughi S., Deb Roy. Tweet Acts: A Speech Act Classifier for Twitter, ICWSM'16, 17–20 May, Cologne, Germany. In Proceedings of the 10th AAAI Conference on Weblogs and Social Media (ICWSM 2016). Cologne, Germany.

[32] Ratkiewicz J, Conover M., Meiss M., Gonc¸alves B., Patil S., Flammini A., Menczer F. Detecting and tracking the spread of astroturf memes in microblog streams. *arXiv*:1011 3768, 2010.

[33] Black W.J., Procter R., Gray S., Ananiadou S. 2012. A data and analysis resource for an experiment in text mining a collection of micro-blogs on a political topic. Proceedings of the Eighth International Conference on Language Resources and Evaluation. Istanbul. (see Behind the rumours: how we built our Twitter riots interactive, https://www.theguardian.com/news/datablog/2011/dec/08/twitter-riots-interactive)

[34] De Domenico M., Lima A., Mougel P., Musolesi M. 2013 The Anatomy of a Scientific Rumor. *Scientific Reports*, 3:2980 doi: 10.1038/srep02980 24135961 10.1038/srep02980 PMC3798885

[35] Lee J., Agrawal M., Rao H.R. 2013 Message diffusion through social network service: The case of rumor and non-rumor related tweets during Boston bombing, *Information Systems Frontiers* 17(5), 997–1005.

[36] Nadamoto A., Mai Miyabe, Eiji Aramaki, Analysis of Microblog Rumors and Correction Texts for Disaster Situations, Proceedings of International Conference on Information Integration and Web-based Applications & Services, 2–4 December 2013, Vienna, Austria.

[37] Starbird, K., Maddock, J., Orand, M., Achterman, P., Mason, R. M. 2014. Rumors, False Flags, and Digital Vigilantes: Misinformation on Twitter after the 2013 Boston Marathon Bombing. In iConference 2014 Proceedings, 654–662. doi: 10.9776/14308

[38] Takayasu M., Kazuya Sato, Yukie Sano, Kenta Yamada, Wataru Miura, Hideki Takayasu. 2015 Rumor Diffusion and Convergence during the 3.11 Earthquake: A Twitter Case Study, *PLoS ONE* 10(4): e0121443 doi: 10.1371/journal.pone.0121443 25831122 10.1371/journal.pone.0121443 PMC4382198

[39] Paavola J., Jalonen, H. 2015. An Approach to Detect and Analyze the Impact of Biased Information Sources in the Social Media. Abouzakhar N. (ed.). Proceedings of the 14th European Conference on Cyber Warfare and Security (ECCWS), 213–219, Univ Hertfordshire, Hatfield, England,

[40] Langston J. 2016. The Twittersphere does listen to the voice of reason—sometimes, 4 4 2016, source: University of Washington (https://article.wn.com/view/2016/04/04/The_Twittersphere_does_listen_to_the_voice_of_reason_sometim/).

[41] Petković T., Z Kostanjčar, P Pale. 2005. E-mail system for automatic hoax recognition. XXVII. International Convention MIPRO 2005 Bd. CTS & CIS, Opatija, Croatia, pp. 117–121, ISBN 953–233–012–7.

[42] Vuković M, Krešimir Pripužić, Hrvoje Belani. 2009. An intelligent automatic hoax detection system. Knowledge-Based and Intelligent Information and Engineering Systems, volume 5711 of the series Lecture Notes in Computer Science, 318–325.

[43] Chen Yoke Yie, Suet-Peng Yong, Adzlan Ishak. 2014 Email Hoax Detection System Using Levenshtein Distance Method. *JCP* 9(2), 441–446.

[44] Collier N., Doan S., Kawazoe A., Goodwin R.M., Conway M., Tateno Y., Ngo Q.H., Dinh D. (Dinh Dien), Kawtrakul A., Takeuchi K. 2008 BioCaster: detecting public health rumors with a Web-based text mining system. *Bioinformatics*, 24(24), 2940–2941. doi: 10.1093/bioinformatics/btn534 18922806 10.1093/bioinformatics/btn534 PMC2639299

[45] Zubiaga A., Aker A., Bontcheva K., Liakata M. and Procter R. Detection and resolution of rumours in social media: A survey. arXiv preprint arXiv:1704 00656, 2017.

[46] Yang Fan, Yang Liu, Xiaohui Yu, Min Yang. Automatic detection of rumor on Sina Weibo, Proceedings of the ACM SIGKDD Workshop on Mining Data Semantics, p. 1–7, 12–16 August 2012, Beijing, China.

[47] URL-Weibo http://www.weibo.com/.

[48] Resnick P., Samuel Carton, Souneil Park, Yuncheng Shen, Nicole Zeffe. 2014. Rumor Lens: A System for Analyzing the Impact of Rumors and Corrections in Social Media, Computation+Journalism Symposium 2014—Columbia University, New York, USA.

[49] Seo E., Prasant Mohapatrab, Tarek Abdelzaher. Identifying Rumors and Their Sources in Social Network, *Proc*. *SPIE* 8389, Ground/Air Multisensor Interoperability, Integration, and Networking for Persistent ISR III, 83891I (1 5 2012), doi: 10.1117/12.919823

[50] Shah D., Zaman T. 2011 Rumors in a network: Who’s the culprit? *IEEE Transactions on Information Theory* 57(8), 5163–5181.

[51] Newman M.E.J. 2002 The spread of epidemic disease on networks, *Phys*. *Rev*. *E*, 66, 016128. 10.1103/PhysRevE.66.016128 12241447

[52] Zhao Laijun, Jiajia Wang, Rongbing Huang. 2015 Immunization against the Spread of Rumors in Homogenous Networks, *PLoS ONE* 10(5): e0124978 doi: 10.1371/journal.pone.0124978 25933430 10.1371/journal.pone.0124978 PMC4416730

[53] Bordia P., DiFonzo N. 2004 Problem solving in social interactions on the Internet: Rumor as social cognition. *Social Psychology Quarterly*, 67(1), 33–49.

[54] URL-TwitterTrails http://twittertrails.wellesley.edu/

[55] Finn S., Metaxas P.T., Mustafaraj E. 2014. Investigating Rumor Propagation with TwitterTrails. Computation and Journalism Symposium, Columbia University, New York.

[56] Fang J.Jin F., Wang W., Zhao L., Dougherty E., Cao Y., Lu C.-T., Ramakrishnan N. 2014 Misinformation propagation in the age of twitter. *Computer*, 47(12), 90–94.

[57] Granovetter MS (1973). The strength of weak ties. American Journal of Sociology, 78(6), 1360–1380. doi: 10.1086/225469

[58] Acemoglu D., Ozdaglar A., ParandehGheibi A. 2010 Spread of Misinformation in Social Networks, *Games and Economic Behavior* 70, 194–227.

[59] Menczer F. 2016. The Spread of Misinformation in Social Media. In Proceedings of the 25th International Conference Companion on World Wide Web (WWW '16 Companion). International World Wide Web Conferences Steering Committee, Republic and Canton of Geneva, Switzerland, 717–717.

[60] Silverman C. 2016. Emergent: A real-time rumor tracker (last access 21017) http://emergent.info

[61] Turenne N. 2016 Analyse de données textuelles sous R (Text Data Analytics with R), ISBN: 978-1-78405-107-, ISTE publisher, London, United-Kingdom, 318 pp.

[62] URL-Twitter https://twitter.com/search-advancedhttp://www.twitter.com/.

[63] Bernard P., Lecomte J., Dendien J., Pierrel J.M. 2002. Computerized linguistic resources of the research laboratory ATILF for lexical and textual analysis: Frantext, TLFi, and the software Stella 3rd International Conference on Language Resources and Evaluation, Las Palmas, Canary Islands, Spain.

[64] Gaiffe B., K. Nehbi. 2009. TEI Est Républicain. (date accessed 2017) http://www.cnrtl.fr/corpus/estrepublicain/

[65] Burnard L. 2000. The British National Corpus Users Reference Guide. (date accessed 2017) URL: http://www.natcorp.ox.ac.uk/docs/userManual/

[66] Davies M., The Corpus of Contemporary American English (COCA): 400+ million words, 1990-present (2008). Available at http://www.americancorpus.org. (date accessed 2017) (http://corpus.byu.edu/coca/).

