# Supplementary material for: The rumour spectrum
Source: PLoS One. 2018 Jan 19;13(1):e0189080. doi: 10.1371/journal.pone.0189080 (PMC5774683; doi:10.1371/journal.pone.0189080)
Supplement: S1 Appendix — (DOCX) [file pone.0189080.s001.docx]

**Features**

Hereafter we summarise the features that we can find in the previous, cited studies. We can distinguish six mains categories of 53 features:

- Linguistic Features pertain to the microblog message text
- User Features describe the characteristics of an individual user
- Network Features pertain to either the message propagation or friendship network
- Temporal Features look at the time stamps of the messages
- Physical IP, location or used software
- Multibase Use of other database to correlate information

In this sense, not all categories are available for any social media platform. For instance, detecting hoaxes on Wikipedia cannot be modelled by temporal information, but for Twitter it is relevant.

**Linguistic category**

Table 9. Linguistic features

**User category**

Table 10. User features

**Network category**

Table 11. Network features

**Temporal category**

Table 12. Temporal features

**Physical category**

Table 13. Physical features

**Multibase category**

Table 14. Multibase features

For some features, aggregated features can be inferred easily for integration in models, such as per cent of messages having hashtags, or average score of sentiment for a corpus. Since most of the big social media platforms are private, not all data are available (features or messages). For instance, the IP feature in Twitter is not available, and messages are only available for the previous seven days. On Wikipedia a repost is available in terms of URL mentions, but user information about the modification page is not reachable. In all results, on average, the linguistic and network features category plays an important role, and second is the temporal category.
