# Supplementary material for: The rumour spectrum
Source: PLoS One. 2018 Jan 19;13(1):e0189080. doi: 10.1371/journal.pone.0189080 (PMC5774683; doi:10.1371/journal.pone.0189080)
Supplement: S2 Appendix — (DOCX) [file pone.0189080.s002.docx]

**Twitter API**

Hereafter we present a sample of code with R language to build a random corpus of tweets. For other corpora it just requires other keywords. Rumorous corpora are more difficult to build with API because Twitter does not allow extraction before 7 days back in time at the moment of extraction so we used the advanced search user interface and basic copy/paste (see <https://twitter.com/search-advanced?lang=en>).

About rumorous corpora these are for each corpus a specific query :

1. Holland corpus : (hollande AND hidalgo AND fils) lang:fr
2. Lemon corpus : (citron AND cancer) lang:fr
3. Pin corpus : (pin AND atm AND police) lang:en
4. Swine corpus : (“swine flu”AND “South Africa”) lang:en

About event tweet corpora these are for each corpus a specific query :

1. Rihanna French : (Rihanna AND concert AND 2016) lang:fr
2. Rihanna English : (Rihanna AND concert AND 2016) lang:en
3. Euro 2016 French : (UEFA AND euro AND 2016) lang:fr
4. Euro 2016 English : (UEFA AND euro AND 2016) lang:en

This is the code that can be used for random rumorous corpus building (just replace by your personal API secret keys):

install.packages("twitteR")

library(twitteR)

ConsumerKey <- "YourKey"

ConsumerSecret <- "YourSecret"

AccessToken <- "YourToken"

AccessTokenSecret <- "YourTokenSecret"

setup_twitter_oauth (ConsumerKey, ConsumerSecret, AccessToken, AccessTokenSecret)

random.tweets = searchTwitter( "and", lang="en", n=1000 )

df <- do.call("rbind", lapply(random.tweets, as.data.frame))

write.csv(df$text, file=”path/to/corpus", fileEncoding = "UTF-8")

CorpusRandomFr <- read.csv( file="path/to/corpus", encoding = "UTF-8" );

CorpusRandom = CorpusRandomFr[[2]]
